# 628. Geographic distribution of hospitalized severe malaria cases in the United States, 2012–2018

**DOI:** 10.1093/ofid/ofae631.193

**Published:** 2025-01-29

**Authors:** Tyler S Brown, Kimberly E Mace, Satoshi Koiso, Alison Ridpath, Eren Gulbas, Alison T Walker, Regina C LaRocque, Seymour Williams, Edward T Ryan, Emily P Hyle

**Affiliations:** Boston University Chobanian & Avedisian School of Medicine, Boston, MA; Malaria Branch, Division of Parasitic Diseases and Malaria, National Center for Emerging and Zoonotic Infectious Diseases, the U.S. Centers for Disease Control and Prevention, Atlanta, Georgia; Medical Practice Evaluation Center, Massachusetts General Hospital, Boston, Massachusetts; Centers for Disease Control and Prevention, Atlanta, Georgia; Massachusetts General Hospital, Boston, Massachusetts; Centers for Disease Control and Prevention, Atlanta, Georgia; Massachusetts General Hospital, Boston, Massachusetts; Malaria Branch, Division of Parasitic Diseases and Malaria, National Center for Emerging and Zoonotic Infectious Diseases, the U.S. Centers for Disease Control and Prevention, Atlanta, Georgia; Massachusetts General Hospital, Boston, Massachusetts; Massachusetts General Hospital, Boston, Massachusetts

## Abstract

**Background:**

Between 200–300 severe malaria cases are diagnosed annually in the U.S. Intravenous artesunate (IVAS), the FDA-approved 1st-line therapy for severe malaria since 2020, is commercially available but at high cost. Uncertainty regarding where people with severe malaria will seek medical care poses supply chain challenges. We identified hospitals where people with severe malaria were treated and mapped proximity to Level 1 trauma centers as a proxy for hospitals that provide complex clinical care, which could assist with planning for IVAS distribution.

Location identification and manual adjudication process for hospitals that hospitalized people with severe malaria as reported to CDC from 2012–2018.

**Figure:**
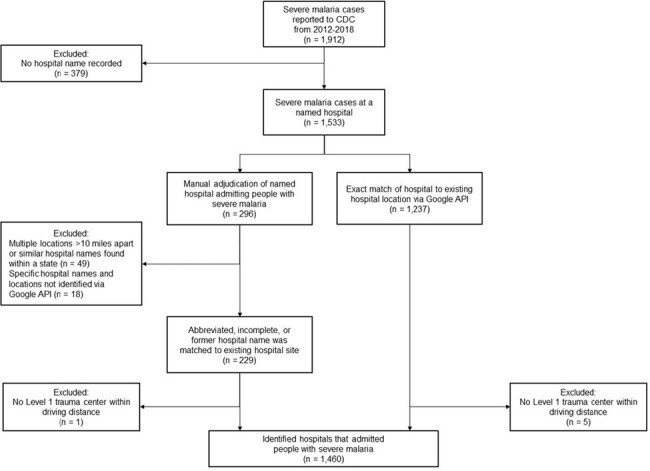

Of the original 1,912 cases of severe malaria reported to the CDC from 2012-2018, we excluded 379 cases that were not admitted or were admitted but missing a hospital name. Of the remaining 1,533 severe malaria cases that were eligible for adjudication, 1,237 were an exact match with an existing hospital location. Of the 296 cases that were not an exact match, we manually adjudicated 229 cases by clarifying a hospital name that was abbreviated, incomplete, or had a change in name; only 49 cases could not be identified given multiple possible sites with the same name, and 18 cases with no name that matched a hospital site. Six additional cases were excluded because of occurring at a site with no Level 1 trauma center within driving distance (i.e., Alaska and US Virgin Islands).

**Methods:**

Using 2012–2018 data reported to the Centers for Disease Control and Prevention (CDC), we analyzed free text of U.S. hospital names where people were treated as inpatients for severe malaria. We identified these hospitals using Google Maps Geocoding Application Programming Interface (API) followed by manual adjudication. We excluded cases when data were incomplete or ambiguous or if a Level 1 trauma center was inaccessible (e.g., Alaska or U.S. Virgin Islands). We then mapped hospitals to U.S. counties and estimated travel times between hospitals and the nearest Level 1 trauma center as per 2014–2016, using Google Maps Distance Matrix API.

Average numbers of severe malaria cases at identified hospitals per US county annually as reported to the CDC during 2012–2018.

**Figure:**
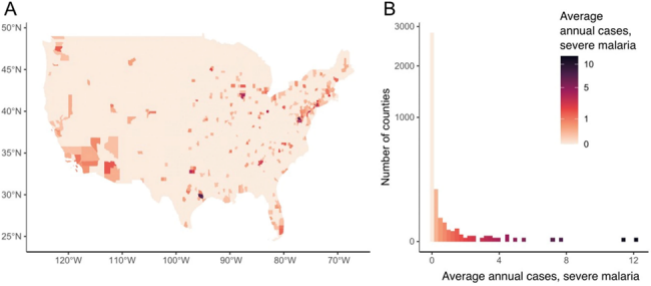

Panel A shows the geographic distribution and average number of severe malaria cases at identified hospitals per year by county reported to CDC during 2012–2018; darker color shows counties with more severe malaria cases reported. Panel B shows that the distribution is highly skewed to 2 counties that reported more than 10 inpatient cases annually; 2,824 counties (pale peach) reported no cases of severe malaria admitted to an identified hospital during 2012–2018.

**Results:**

After excluding 379 cases with no recorded hospital name from 1,912 severe malaria cases, we successfully adjudicated hospitals for 1,460/1,533 cases (Fig 1). Two counties reported >10 inpatient cases per year (Bronx County, NY and New York County, NY), and 2,824 counties never reported a hospitalized severe malaria case (Fig 2). Almost 35% of inpatient cases occurred at Level 1 trauma centers (508/1,460 [34.8%]) (Fig 3). Of the 952/1,460 (65.2%) inpatient cases not at Level 1 trauma centers, 891/952 (93.5%) were at hospitals located < 2h estimated driving time of a Level 1 trauma center. Only 61/1,460 (4.2%) cases were at locations >2h from a Level 1 trauma center. Limitations include that the analysis did not consider the original site of clinical presentation with malaria or locations of people with severe malaria not hospitalized.

Histogram of modeled travel times between each severe malaria case at an identified hospital and the nearest Level 1 trauma center.

**Figure:**
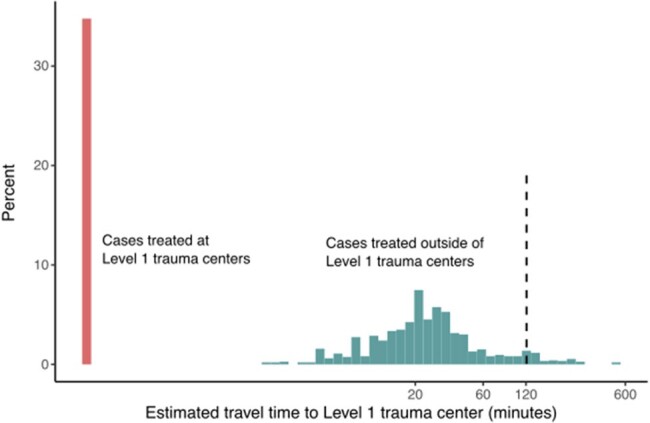

Of severe malaria cases reported to CDC in 2012–2018 and hospitalized at an identified hospital, 34.8% were treated at Level 1 trauma centers (red bar); for severe malaria cases that were not at Level 1 trauma centers, we found that modeled transit times between treatment site and Level 1 trauma center were less than 2 hours except in 4.2% of severe malaria cases at an identified hospital (right of black dashed vertical line).

**Conclusion:**

More than 95% of people admitted for severe malaria at identified hospitals reported to the CDC in 2012–2018 were at or within 2h estimated driving time of a Level 1 trauma center, which may suggest priorities for implementing IVAS distribution.

**Disclosures:**

**Alison Ridpath, MD, MPH**, Abbvie Inc.: Stocks/Bonds (Public Company)|Amarin Corperation PLC: Stocks/Bonds (Public Company)|Amgen Inc: Stocks/Bonds (Public Company)|BioNTech: Stocks/Bonds (Public Company)|Immunic Inc: Stocks/Bonds (Public Company)|infinity Pharmaceutical Companies: Stocks/Bonds (Public Company)|IQVIA Holdings Inc: Stocks/Bonds (Public Company)|Johnson and Johnson: Stocks/Bonds (Public Company)|Merck and Co inc: Stocks/Bonds (Public Company)|Pfizer Inc.: Stocks/Bonds (Public Company)|Protagonist Therapeutics: Stocks/Bonds (Public Company)

